# Knowledge Graph Based Hard Drive Failure Prediction

**DOI:** 10.3390/s22030985

**Published:** 2022-01-27

**Authors:** Tek Raj Chhetri, Anelia Kurteva, Jubril Gbolahan Adigun, Anna Fensel

**Affiliations:** 1 Semantic Technology Institute (STI), Department of Computer Science, University of Innsbruck, 6020 Innsbruck, Austria; anelia.kurteva@sti2.at (A.K.); or anna.fensel@wur.nl (A.F.); 2 Quality Engineering (QE-Lab), Department of Computer Science, University of Innsbruck, 6020 Innsbruck, Austria; jubril.adigun@uibk.ac.at; 3 Wageningen Data Competence Center, Wageningen University & Research, 6708 PB Wageningen, The Netherlands; 4 Consumption and Healthy Lifestyles Chair Group, Wageningen University & Research, 6706 KN Wageningen, The Netherlands

**Keywords:** hard drive, failure prediction, knowledge graphs, machine learning, predictive maintenance, reliability

## Abstract

The hard drive is one of the important components of a computing system, and its failure can lead to both system failure and data loss. Therefore, the reliability of a hard drive is very important. Realising this importance, a number of studies have been conducted and many are still ongoing to improve hard drive failure prediction. Most of those studies rely solely on machine learning, and a few others on semantic technology. The studies based on machine learning, despite promising results, lack context-awareness such as how failures are related or what other factors, such as humidity, influence the failure of hard drives. Semantic technology, on the other hand, by means of ontologies and knowledge graphs (KGs), is able to provide the context-awareness that machine learning-based studies lack. However, the studies based on semantic technology lack the advantages of machine learning, such as the ability to learn a pattern and make predictions based on learned patterns. Therefore, in this paper, leveraging the benefits of both machine learning (ML) and semantic technology, we present our study, knowledge graph-based hard drive failure prediction. The experimental results demonstrate that our proposed method achieves higher accuracy in comparison to the current state of the art.

## 1. Introduction

In recent years, advancements in fields such as machine learning (ML) have led the shift towards the fourth industrial revolution, also referred to as Industry 4.0 [1]. Its main objective is to *“bring an increase in productivity in both production and management systems”* [2] by focusing on analytics-driven insight development such as predictive maintenance (PdM) [3]. The transition to Industry 4.0 is driven by the Internet of Things (IoT) and the amount of generated data, which has increased exponentially, requiring large data centres being used to meet companies’ storage demands [4]. The data itself is stored on hard drives (HDs), which are one of the most commonly used data storage devices [5]. However, HDs are often prone to different failures. The most common failures can be categorised as logical, mechanical, or firmware failures (https://www.salvagedata.com/common-causes-of-hard-drive-failure/ (accessed on 6 June 2021)). Such failures can result in system unavailability or even permanent data loss, which can have a negative impact due to system downtime and can lead to monetary losses for companies [6]. For example, 78% of hardware replacements in Microsoft (https://www.microsoft.com/de-at/ (accessed on 10 June 2021)) data centres were due to HD failure [7]. HD reliability is key and the research for solutions that can predict such failures is an ongoing endeavour.

Through the years, various ML and reasoning approaches based on algorithms such as Decision Trees (DT) [8], k-Nearest Neighbour [9], Classification Trees (CT) [10], and Regression Trees (RT) [11] have been adopted successfully for PdM (i.e., the failure prediction accuracy rates have improved) [12,13,14]. State of the art solutions such as [7,15,16,17,18] have shown to achieve more than 90% accuracy in failure prediction. However, ML on its own lacks context awareness [19] and therefore, lacks the benefits that comes with it. The term context refers to any information that can be used to characterise an entity’s situation (i.e., whether a person, place, or object) [20]. For example, let us consider the following knowledge graph (KG) facts as triples *(“Humans”, “are”, “mortal”)* and *(“Socrates”, “is a”, “human”)*. Now, using these triples we can deduce the fact that *(“Socrates”, “is”, “mortal”)*. This is made possible by the context-awareness generated as a result of the connected relationships. However, as machines do not inherently have the deductive capability, we need to formalise the logical consequences based on entailment regimes (https://www.w3.org/TR/sparql11-entailment/ (accessed on 12 August 2021)) to make such deductions, which ontologies and KGs are capable of due to their ability to model relationships [21]. In the case of the hard drive failure prediction we can have similar benefits of KGs. For example, let us consider the following KG triples *(“Hard drive”, “smart5”, “10”)*, *(“Hard drive”, “has humidity”, “89”)*, *(“Hard drive”, “storage has average temperature”, “65”)*. Now, using these KG triples we can deduce the fact that *(“Hard drive”, “status”, “fail”)* (i.e., hard drive will fail). This is due to the fact that high humidity and temperature deteriorate hardware, particularly electronic devices [22]. In ML, we can leverage such advantages of the KGs using the nodes (or entities) representation and taking features of a local neighbourhood coupled with relationships (details in Section 4) to improve the prediction. Therefore, considering the benefits of using KGs and extending our initial idea (see [23]), we hypothesise that by combining KGs and ML, we can achieve even greater benefits in failure prediction and present our work on hard drive failure prediction. The proposed approach takes the benefits of both: KGs’ context-awareness and ML pattern learning and predictability capabilities. It also eliminates the limitations of rule-based approaches, which requires us to have all the rules defined beforehand and also lacks predictability like ML. Recent studies, such as [24], have also highlighted such limitations.

KG, which Fensel et al. [25] define as *“very large semantic nets that integrate various and heterogeneous information sources to represent knowledge about certain domains”*, have the ability to transform data into information and information into knowledge by creating meaningful relationships between entities [21,25]. With the help of relationships, a KG is able to provide context to ML. Other benefits of semantic technology such as data interopretability, connectivity of data across domains and faster and easier knowledge discovery, have been further discussed in more detail in [26,27,28,29,30]. Because of the benefits of semantic technology, we can find its application in domains such as predictive maintenance [31,32] and recommender systems [33] that utilise ontologies [34] and KGs. Additionally, Kainzner et al. [35] demonstrate the potential benefits of semantic technology in relevant domains such as manufacturing. Other than the relationships, KGs also offer a simple and adaptable way to include additional information. For example, humidity can have a significant impact on HD failure [22]. However, such environmental data is not available in SMART (Self-Monitoring, Analysis, and Reporting) (https://www.ibm.com/support/pages/define-smart-used-smartcollect (accessed on 13 June 2021)) attributes. SMART attributes are obtained from SMART technology [36], which enables monitoring of the hard disk’s status (see [7]) and reporting of various reliability indicators.

In this paper, we demonstrate the value of combining KGs and ML for predicting HD failures. Our proposed approach uses KGs and relational graph convolutional networks (RGCN), a ML technique for failure prediction (details in Section 4.4). The KG used in this study is based on data from SMART attributes.

The paper is structured as follows. Section 2 presents an overview of related works on hard drive failure prediction. Section 3 provides information on the following methodology. A detailed description of the proposed solution in the paper is presented in Section 4, while its performance evaluation is described in Section 5. Conclusions can be found in Section 6.

## 2. State of the Art

This section summarises the related work. The use of both ML techniques and semantic technology can be found in various domains such as failure prediction in manufacturing. However, because our study is focused on the prediction of HD failure, we restrict our review of related work to HD. Schoenfisch et al. [37], for example, used semantic technology (ontology) and Markov logic networks (see [38]) to conduct a study on the root cause analysis of information technology (IT) infrastructure. Such studies are not included in our analysis because they do not address HD failure prediction directly. The first section, Section 2.1, summarises the study using semantic models, while the second section, Section 2.2 focuses on the studies with ML techniques. Finally, Section 2.3 summarises the related work presented in Section 2.1 and Section 2.2.

### 2.1. Semantic Model-Based Study

Mamoutova et al. [24] present an ontology-based approach to automatic analysis of data storage systems log files extending their work, a knowledge-based approach for enterprise storage system diagnosis using ontology [39]. Their work incorporates expert knowledge stored in a knowledge base as RDF (Resource Description Framework)/XML (Extensible Markup Language) format by converting it to the N-Quads format of the graph database. Mamoutova et al. [24] use an ontology to represent fault symptoms such as damage due to abnormal temperature change, which are then reasoned over using monitored data for diagnostic purposes. SPARQL (https://www.w3.org/TR/rdf-sparql-query/ (accessed on 3 August 2021)) and GraphQL+ (https://docs.dgraph.io/query-language (accessed on 3 August 2021)) are used to facilitate diagnosis querying. As the authors point out, the limitation is that each fault that needs to be detected must be described by the combination of values and the used monitoring parameters. Furthermore, such an approach is limited in its ability to express abnormal values of a parameter in terms other than a simple threshold or binary value, such as a bounded interval. Mamoutova et al. [24] also use ML-based techniques such as random forest (RF), long-short-term memory (LSTM), gated recurrent unit (GRU), and LSTM with attention in a complex situation where an ontology-based approach fails. This demonstrates the limitations of a semantic-only approach even more.

### 2.2. Machine Learning-Based Study

Su et al. [40] use the RF classification algorithm and SMART attributes to conduct a study on hard drive predictive maintenance. The solution in [40] has the ability to make real-time predictions by utilising technologies such as Apache Hadoop (https://hadoop.apache.org (accessed on 2 June 2021)) and Apache Spark (https://spark.apache.org (accessed on 6 September 2021)). The use of a RF classification algorithm for HD failure prediction can also be seen in [41,42]. However, in addition to using the same ML algorithm, Shen et al. [41] use a sliding window to reduce the effect of noise and an additional part voting strategy to improve the prediction accuracy. On the other hand, Züfle et al. [42] use additional techniques such as synthetic minority oversampling technique (SMOTE) [43] and enhanced structure-preserving oversampling (ESPO) [44] together with random forest. Similar to Su et al., Züfle et al. and Shen et al., Mashhadi et al. [45] also make use of RF algorithm. Mashhadi et al. conducted a case study of the HD time to failure using SMART attributes in cloud manufacturing settings. The authors perform regression analysis with the RF classification algorithm to predict the time to failure. Further, the authors present findings of specific SMART attributes, their importance and how they correlate with each other across different brands. For example, it has been discovered that higher SMART attribute values, such as SMART 5, SMART 10, and SMART 187, have a strong correlation with failures. The other study, by Li et al. [5], do not use a RF algorithm, but rather a tree-based algorithm based on decision trees (DTs) [8] and gradient boosting regression trees (GBRT) [46]. The DTs was used for failure prediction, while the GBRT was used for health degree evaluation (i.e., a value set for each failed sample between [−1, 0]). Furthermore, a simple voting strategy with DT is used to improve prediction, and the work is evaluated using the simulated scenario.

Han et al. [47] use a streaming approach to predict disc failure with the help of incremental learning algorithms such as DT. The authors follow an ensemble approach combining multiple DTs to overcome limitations such as single DT’s diversity and look-ahead. SMART attributes are also used in the work to predict failure. Similar to Han et al., Ganguly et al. [48] also used an ensemble approach for their study. Ganguly et al. conducted a study on hard drive failure prediction with the Azure (https://azure.microsoft.com/en-us/ (accessed on 12 September 2021)) cloud platform using a two-step ensemble method, which employ a non-parametric DT at stage 1 and logistic regression (LR) at stage 2. The solution in [48] has been put into production and it has shown to reduce the downtime of virtual machines (VMs) caused by HD.

Studies by Liu et al. [6], Zang et al. [49], and Santo et al. [50], on the other hand, use a deep learning approach for hard drive failure prediction. The work of Liu et al. focuses on cloud storage systems and applied modified recurrent neural networks (RNN). The main difference (in comparison to previous research) is how the current hidden layer is updated, which is by feeding the previous time step hidden layer and output layer. Similarly, the work of Santo et al. used LSTM, RNN based deep learning technique, while the work of Zang et al. make use of adversarial training [51] with SMART attributes. Both the work of Santo et al. and Zang et al. focus on hard drive health prediction. In contrast to previous research, Franklin [52] conducted an empirical study on hard drive failure prediction and observed an increase in the reallocated sector count of one of the SMART attributes prior to a failure.

### 2.3. Summary

In conclusion, the majority of the studies discussed in Section 2 apply ML techniques or, more recently, a few techniques based on semantic models for HD PdM failure prediction. We present a summary of the existing solutions in Table 1. The table presents each study, the main method that has been used (ML-based or semantic-based), and, when available, performance and training time statistics and current limitations. While most of the presented solutions achieve accuracy of failure prediction above 90% and use SMART attributes, context-awareness is rarely addressed. From the presented solutions, only the one by Mamoutova et al. [24] uses semantic technology, namely ontologies, to provide context to the selected ML model.

Two of the main limitations of the existing ML solutions as presented in Section 2 and Table 1 is the lack of context awareness (e.g., why and how a failure occurred, how the failure affects the HD overtime) and standardisation when dealing with heterogeneous data types. The numerous ongoing studies demonstrate that the problem of predicting HD failure requires a solution that takes into account the changing technological landscape and advancement.

All these limitations can be resolved with the help of semantic technologies such as ontologies and knowledge graphs, which provide both context and a unified data model in machine-readable format. The use of semantic technology, namely knowledge graphs, enables context-awareness through the relationships that hold between concepts (in a specific domain) as demonstrated in studies such as [24]. As a result, in this study, we present our work on combining KGs and machine learning for HD failure prediction.

## 3. Methodology

**Figure 1:**
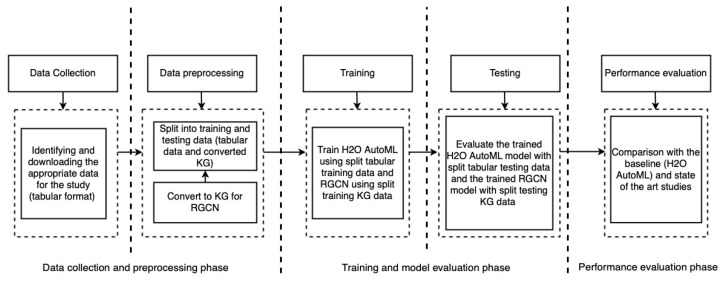
High level overview of followed methodology.

Figure 1 summarises the methodology that we follow. The first phase is data collection, which included the identification of appropriate data (i.e., the selection of a dataset containing HD failure information from the numerous failure datasets available on the internet) and its download. In our case, the downloaded dataset was tabular in nature and was stored as comma-separated value (CSV) files. Section 4.1 contains additional information about the dataset, such as the total number of samples and the location of the collection. The next phase, as shown on Figure 1, is data preprocessing, which consists of the following steps: (i) converting data to KG for RGCN and (ii) splitting data into training and testing sets.

Details on how the tabular data is converted into a KG are presented in Section 4.2. The converted KG serves as an input to RGCN, an ML technique that combines the KG by transforming the KG into low-level representations (details in Section 4.4) and learning patterns, which is also the proposed approach. For example, let us consider the following KG triples: *(“Hard drive”, “smart5”, “10”)*, *(“Hard drive”, “has humidity”, “89”)*, *(“Hard drive”, “storage has average temperature”, “65”)* and *(“Hard drive”, “status”, “fail”)*, which were presented in Section 1 and contains the information about HD failure. To enhance failure prediction, we train the RGCN model on these KG triples containing information about HD failures. Following training, we pass similar KG triples (i.e., KG triples with missing status information) to generate a prediction, which in this case is the status (i.e., failed or working). This approach to RGCN provides additional benefits, such as eliminating the additional step of performing downstream tasks such as classification, that would otherwise be required if embedding techniques such as Rotate3D [53] and K-BERT [54] were used. RGCN is a generalisation of Graph Convolution Networks (GCNs) that operates on relational graph data (or knowledge graphs) [55]. GCNs are a variant of convolutional neural networks (CNNs) on graphs [56]. Moreover, in addition to the details on KG construction from tabular data presented in Section 4.2, we refer to the following studies [57,58] for additional information, as well as study [21] for additional information on KG.

Similarly, during the splitting training and testing data, we split the data into training and testing sets. In this step, we split both the KG and the original tabular data. This is because we also used the H2O (https://www.h2o.ai (accessed on 22 September 2021)) AutoML (https://docs.h2o.ai/h2o/latest-stable/h2o-docs/automl.html (accessed on 22 September 2021)) (Automatic Machine Learning), which provides an automated industrial standard supervised ML algorithm that operates on tabular data [59]. Further, we use it as a baseline for the proposed approach. Section 4.3 provides additional information on H2O AutoML. Further details of the data preprocessing are presented in Section 4.2.

Next, by using the split training data, we perform training in the subsequent step. To train the H2O AutoML, the tabular training data is used, while the KG is used for training the RGCN. Once training is complete, we evaluate the trained H2O AutoML and RGCN models against the split test data. Finally, we perform a performance evaluation by comparing our RGCN results to those of H2O AutoML, which served as a baseline for our RGCN. The model performance is assessed using evaluation metrics (see Section 5.3), which helped us understand how well the model is likely to perform in an unanticipated scenario. In addition to comparing our results to those of H2O AutoML, we make a comparison with the state of the art studies. The results are presented in Section 5.4.

Finally, the steps below summarise the step by step application method for the proposed approach. It differs from the approach of the H2O AutoML, which follows the standard ML approach.

1. The first application requirement is for data in the KGs. If only tabular data is available, it must be transformed into the KGs. Section 4.2, data preprocessing, goes into greater detail about the data transformation process into KGs and Section 4.1 discusses the used data. Additionally, we refer to the studies [57,58] for additional information on converting tabular data to KGs;
2. After obtaining the KGs data, the next step is to divide it into training and testing sets. This step of splitting the data into training and testing set is also common for the H2O AutoML. The task of splitting the data into a training and testing set is performed during the data preprocessing phase;
3. The next step is to train the ML model, which in our case is RGCN, using the split training KG. The details about the training are available in Section 4.4 and Section 5.2;
4. After training is complete, the model is evaluated using split-testing KGs. Section 5.2 and Section 5.4 contain details about the evaluation and its findings, which are performed and obtained aplying evaluation metrics (see Section 5.3).

In the case of H2O AutoML (see Section 4.3), we follow a similar procedure, training, and evaluation, for example, as with RGCN, but with tabular data. Moreover, as an alternative to RGCN, embedding approaches such as Rotate3D [53] and K-BERT [54] can also be used. However, use of such an embedding approaches requires additional ML algorithms to be able to make predictions, as embeddings only transform the KGs into low level representations. This would therefore require more time and effort as one needs to train and optimise multiple algorithms.

## 4. Experiment

In this section, we present details about our experiment. In Section 4.1, we present details about the used data and data preprocessing in Section 4.2. Similarly, in Section 4.3, we present details about H2O AutoML and why we selected it for our study. Finally, in Section 4.4, we provide details on RGCN, such as the reasons for its selection and use in our study. Additionally, we provide details on the experiment, such as the used hyperparameters and activation functions.

### 4.1. Dataset

**Figure 2:**
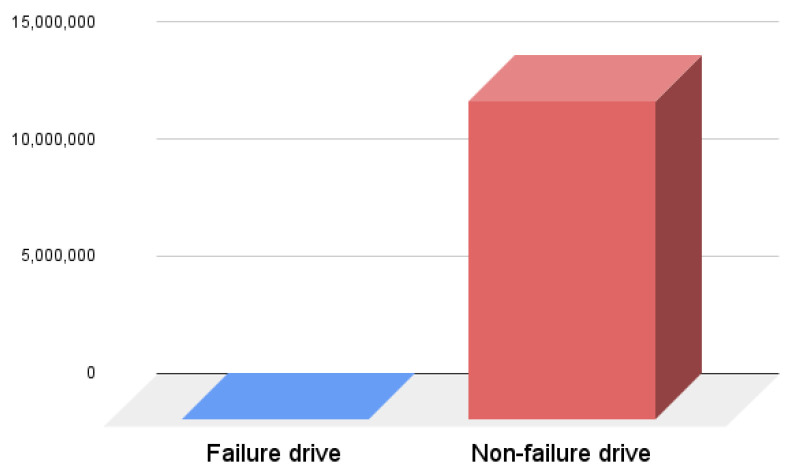
Data distribution.

Our study focuses on HD failure prediction and also uses the ML technique. In order to successfully apply the ML technique, we needed to train ML models. To train ML models, we needed data, so the first step was to gather the dataset about HD failure. In our study, we use data from Backblaze (https://www.backblaze.com/b2/hard-drive-test-data.html (accessed on 10 June 2021)) as it offers a real-world HD failure dataset. The dataset contains information about HD failures represented by SMART attributes. In our experiment, we used the dataset from the third quarter of 2020. The dataset contains a total of 106 SMART attributes of which 53 are raw values and 53 are normalised values. The values are stored in 92 different files, which after download are combined into one CSV file. Figure 2 shows the data distribution after combining the data from all 92 different CSV files. The dataset contains a total of 13,553,809 samples, with only 367 samples representing failed HDs.

### 4.2. Data Preprocessing

**Figure 3:**
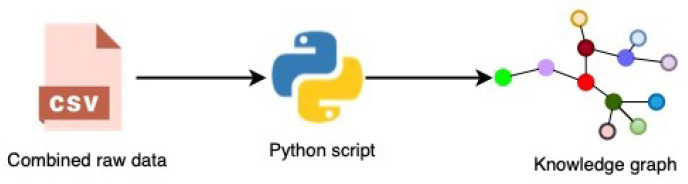
Transforming CSV data into a KG.

The used data consists of 106 SMART attributes (see Section 4.1). Each SMART attribute has a different level of importance. It would be beneficial if we use the attributes that are more important in predicting HD failure. For example, SMART 3, which represents spin-up time, is considered less critical. SMART 5, the other SMART attribute, is considered critical (or more important than SMART 3). If we rely on SMART 3 instead of SMART 5, there is a high chance that our prediction result will not be accurate. As a result, the first data preprocessing step in our study was to fine-tune the SMART attributes based on their significance. Table 2 shows the selected nine SMART attributes based on their importance for HD failure prediction. The significance of the SMART metrics was determined using information obtained from manufacturers, such as Segate (https://www.seagate.com/gb/en/ (accessed on 23 September 2021)), and a review of the literature. Once we have finalised the SMART attributes that are to be used in our study, the next step is to convert the data into KGs. In case of H2O, we use tabular data with selected SMART attributes (see Table 2). Figure 3 presents the process of creating the KG.

**Figure 4:**
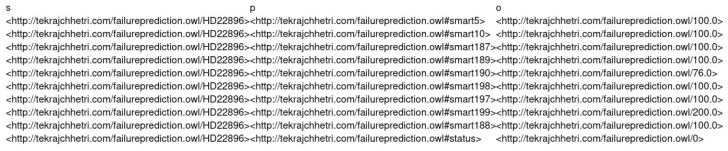
KG stored in CSV.

**Figure 5:**
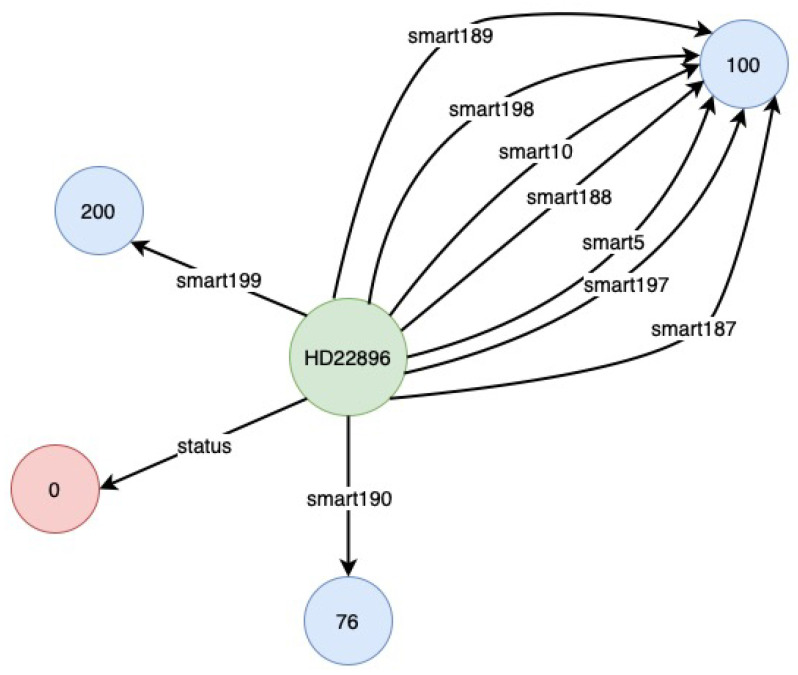
Visualisation of a KG sample—I.

**Figure 6:**
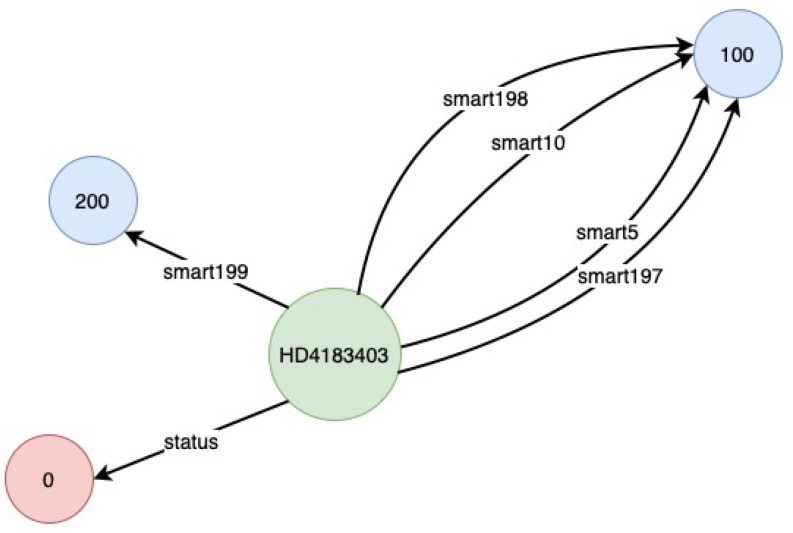
Visualisation of a KG sample—II.

A Python script is used to transform the combined raw data into a KG, which we implemented. For the transformation of raw data into KGs, we followed the principles of RDF Mapping Language (RML) (https://rml.io (accessed on 24 September 2021)). After the transformation, the raw CSV data is saved in CSV format again, but this time in a triple format (*subject, predicate, object*), denoted by (*s,p,o*) as shown in Figure 4. However, during our experiment, we observed issues such as being out of memory and slow processing when using the KGs stored in CSV. Therefore, to deal with these issues, we transformed the KGs stored in CSV to NetworkX (https://networkx.org (accessed on 24 September 2021)) representation. The NetworkX representation allowed us to represent KGs as a graph, which can be directly fed to RGCN. Similarly, Figure 5 and Figure 6 show the visualisation of an instance of the created KGs. We have removed the Uniform Resource Identifier (URIs) from Figure 5 and Figure 6 to simplify the visualisation.

One can observe the difference in the number of relationships and nodes in Figure 5 and Figure 6. This is due to missing values in the original data. In the case of the KG instance depicted in Figure 5, there are no missing values, thus all 10 relationships are present. In addition to the nine relationships discussed before, one extra relationship represents the status of the hard drive based on the SMART attributes value. However, in the KG example depicted in Figure 6, there were missing values, resulting in the absence of some relationships and the corresponding nodes (or tail). This is also an advantage in this case as we can ignore the missing values, which would otherwise have to be filled using imputation techniques.

### 4.3. H2O AutoML

H2O AutoML is a fully automated supervised ML algorithm that is part of the H2O framework [59]. H2O is an open-source, distributed ML platform that is designed to scale large datasets and to produce high-quality models, which are suitable for enterprise deployment [59]. Our work uses H2O due to its ability to provide high-quality, enterprise-ready deployment models. Additionally, we consider H2O AutoML because it works with only tabular data (i.e., not KGs) and is the state of the art technique that serves as the baseline for our KGs-based approach.

**Figure 7:**
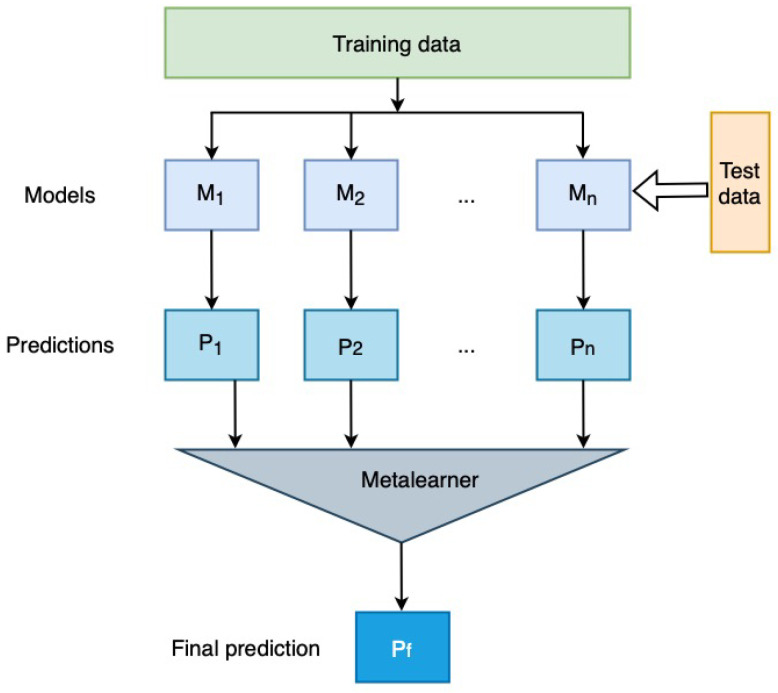
Stacked ensemble.

H2O AutoML provides an implementation for different state of the art algorithms. The implemented base ML algorithms in H2O AutoML include XGBoost Gradient Boosting Machines (XGBoost) (https://docs.h2o.ai/h2o/latest-stable/h2o-docs/data-science/xgboost.html (accessed on 27 September 2021)), Gradient Boosting Machine (GBM) (https://docs.h2o.ai/h2o/latest-stable/h2o-docs/data-science/gbm.html (accessed on 27 September 2021)), Random Forest (RF) (http://docs.h2o.ai/h2o/latest-stable/h2o-docs/data-science/drf.html (accessed on 27 September 2021)), Deep Neural Networks (DNN) (http://docs.h2o.ai/h2o/latest-stable/h2o-docs/data-science/deep-learning.html (accessed on 27 September 2021)) and Generalised Linear Model (GLM) (http://docs.h2o.ai/h2o/latest-stable/h2o-docs/data-science/glm.html (accessed on 27 September 2021)). Further, H2O AutoML provides Stacked Ensembles (http://docs.h2o.ai/h2o/latest-stable/h2o-docs/data-science/stacked-ensembles.html (accessed on 27 September 2021)) algorithms. Stacked ensembles, also known as stacking or super learning, allow us to improve the prediction accuracy by training a *metalearner* on ensemble model predictions, as shown in Figure 7. Stacking ensembles, as opposed to ensemble learning, which takes the weakest learner (such as a decision tree), considers the strongest learned models. H2O uses all models ensemble and best of family models for stacking. The “all models” ensemble contains all the models while the best of the family ensemble only includes the best performing model from each algorithm family [59].

In our experiment with H2O, we used four different algorithms: stacked ensembles, GLM, distributed random forest (DRF), and XGBoost for training and testing. We excluded DNN due to its implementation being unreproducible (https://docs.h2o.ai/h2o/latest-stable/h2o-docs/automl.html (accessed on 22 September 2021)). The data was divided in three sets: for training, testing, and validation. A total of 70% of the data was set aside for the training set, 15% for the validation set, and 15% for the testing set. The training and testing was performed using the system detailed in Section 5.1. We used a seed value of 123,589,389. The seed value is used to generate pseudo-random numbers, which help achieve reproducibility by producing the same sequence of results as long as we use the same seed value. The algorithms were trained using 6 k-fold cross-validations. The k-fold (i.e., a Monte Carlo [60] method) is a data resampling method used to evaluate the generalisation ability of ML models and to prevent overfitting [61]. It accomplishes this by dividing the data into k subsets of training and validation sets and utilising each fold once for validation and the remaining k − 1 subsets for training. Further, we restricted the number of models to a maximum of 7. H2O also allows us to define a model’s time-bound, which specifies the maximum amount of time a model can run during training. In our case, we used the H2O default time limit of one hour. The parameters used, such as the number of folds in k-fold cross validations and the maximum running time of H2O, were determined through experimentation (i.e., by changing parameters). The presented parameters yielded the better performance (see Section 5.4).

Table 3, Table 4, Table 5 and Table 6 show the tuned hyperparameters of stacked ensemble, GLM, DRF, and XGboost respectively. These hyperparameters were automatically tuned by H2O, which resulted in better performance. As seen in Table 3, we ran 99 iterations of the stacked ensemble model, wherein the chosen metalearner was GLM, using its default setting in H2O. Although, H2O provides a number of different metalearners, we have opted for GLM because it is a flexible algorithm for generalisation of L-base models as well as suitable for prediction tasks. We determine the regularisation as the *Elastic Net Penalty* (with alpha set to 0.5 and this causes lambda result to @ 1.21 × 10${}^{-7}$ as calculated by H2O) to regularise the stacked ensemble model since the latter tends to overfit while combining L base algorithms. In both Table 3 and Table 4, the *logit*, link helps the model to gain further predictive power. We are able to carry out transformations on the predicted probabilities using logit transformation. In addition, as shown in Table 5, we set the number of trees in the forest as 48, although the default number is 50 and the max depth as 20, which is the default provided by H2O—higher values will make the model more complex and may lead to overfitting. We also set the max leaves on each tree to 82. Note that some of the leaves do not get to the max depth of 20 because at that depth, there are about a million leaf nodes to be split with each having multiple columns resulting in millions of split points per tree. These parameters are also displayed as part of the default DRF output on H2O, thus: *Model summary (number of trees, min. depth, max. depth, mean depth, min. leaves, max. leaves, mean leaves)*.

Finally, in Table 6, we highlight the different hyper parameters for the implemented XGBoost algorithm. Here, we see that although XGBoost has four inbuilt tree types, namely *exact, approx, gpu_hist, and hist*, we have opted for the latter, because it is the fastest tree method. This is as a result of the fact that it runs sketching only once while trying to carry out split finding. Additionally, since the booster is of tree type, the learning rate (eta) is kept at the default 0.3, max depth 10 (4 units greater than the default 6), and sample rate in set at 0.6 which falls within the higher range of the limit 0.0 to 1.0 and can help increase training accuracy. Similar to the other implemented algorithms already mentioned, XGBoost is also a powerful gradient boosting machine (GBM) used to solve many problems today.

### 4.4. Relational Graph Convolution Network (RGCN)

RGCN, which was introduced by Schlichtkrull et al. [55] operates on the relational data, taking directed labelled multi-graphs G=(V,R,E) as input. The *V* in graph *G* represent nodes (or entities) $v_{i}\epsilon V$, *E* represents the edge (or relations) represented by the nodes ($v_{i},r,v_{k})\epsilon E$ of type relation rϵR. Graph Convolution Networks (GCNs), which can be thought of as a subset of differential message passing, provide a more accurate representation of nodes by combining connectivity and neighbourhood features, in contrast to DeepWalk or node2vec (https://snap.stanford.edu/node2vec/(accessed on 12 September 2021)), which rely exclusively on connectivity [62,63]. RGCN takes GCN a step further by taking relationships into account while retaining GCN’s advantages, such as advantages of the neighbourhood node. RGCN evaluates Equation (1) while making a neural hidden layer $h_{i}^{l+1}$ update, wherein $N_{i}^{r}$ denotes a set of neighbourhood indices of node *i* under relation rϵR. The *W* in the Equation (1) represents the weight matrix and $c_{i,r}$ and a problem specific normalisation constant. For details on RGCN, we recommend [55]. Furthermore, unlike GCN, RGCN can deal with heterogeneous relationships [64]. As a result, we consider RGCN in our research.

(1) $$h_{i}^{l+1}=\sigma \left(\Sigma_{r\epsilon R}\Sigma_{k\epsilon N_{i}^{r}}\frac{1}{c_{i,r}}W_{r}^{l}h_{k}^{l}+W_{o}^{l}h_{i}^{l}\right)$$

**Figure 8:**
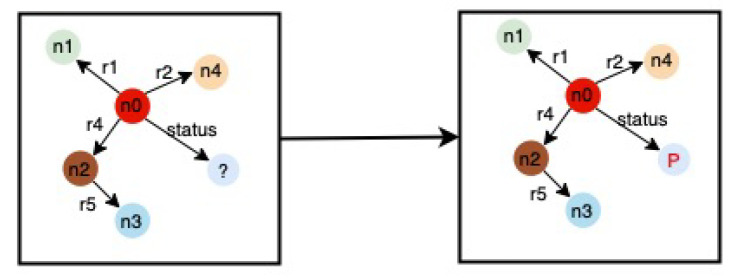
Node classification.

**Figure 9:**
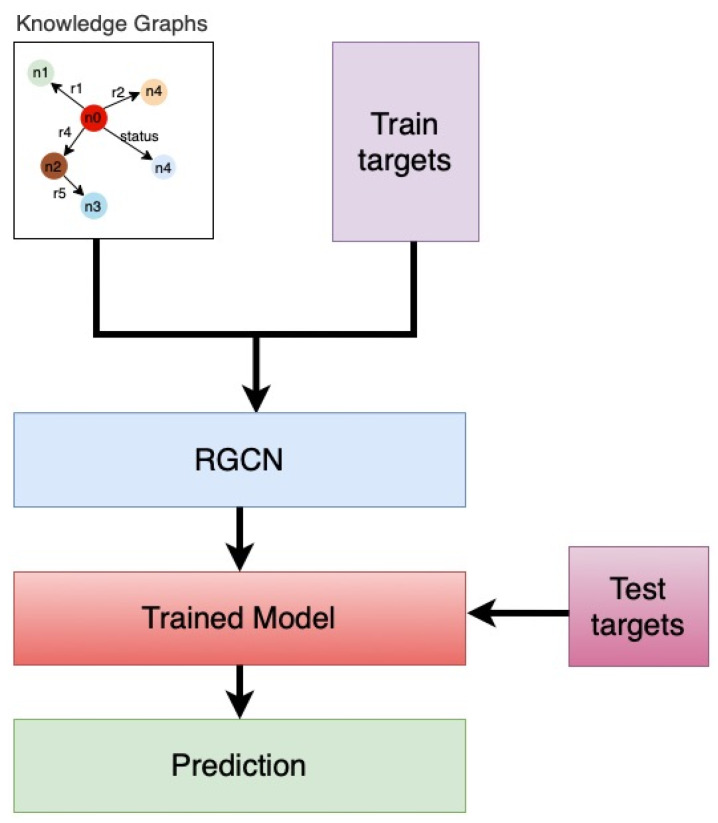
RGCN approach.

The problem of HD failure prediction can be viewed as a node classification task in our study using RGCN, in which we predict the missing node in a KG. In our case, the missing node includes the HD status “fail” or “good”. Figure 8 shows an example of the node classification. As illustrated in Figure 8, we predict the missing node (denoted by *P*) by feeding the KG with missing nodes to the trained model. Figure 9 depicts the detailed approach followed in our study. As illustrated in the Figure 9 method, we begin by training our RGCN model with knowledge graphs and train target labels (or train target). The train target labels contain information about HD failures, which is represented as a node in our KG. Similarly, the test target labels (or test target) include data on HD failures information, which we use to validate our trained model, which we present in Section 5.4. We used 70% of the data in our RGCN study as a training set and 30% as a testing set.

Our RGCN implementation uses four dense layers and one output layer. The first 2 dense layers are made up of 8 hidden units, while the other 2 are made up of 16 hidden units. Experimentation is used to determine the number of hidden units and the density of the layer. Similarly to the H2O case, the presented parameters produced the better result (see Section 5.4) in the case of RGCN. This is because making the model more complex with more parameters leads to overfitting, whereas making the model too simple leads to failure to learn. The activation function is the next most important feature. An activation function distinguishes the neural network from linear models such as linear regression by introducing nonlinearity and assisting the model in learning complex patterns [65]. There are over 20 activation functions available, and choosing the right one is critical [66]. For all of the dense layers in our study, we used the activation function ReLU (https://www.tensorflow.org/api_docs/python/tf/keras/activations/relu (accessed on 27 September 2021)). The reason for this is that ReLU is more resistant to the vanishing gradient problem than other activation functions such as Tanh and Sigmoid [66]. The output layer, however, consists of the sigmoid activation layer. Another critical parameter is the loss function, which quantifies the difference between the prediction and reality. As a result, we always strive to minimise the loss function. Numerous loss functions are available, including mean squared error (MSE) and cross-entropy (CE). The application of loss functions is task-dependent. For instance, the regression problem makes use of the MSE. Due to the classification nature of our task, we chose CE as the loss function. Additionally, there are several variants of CE loss functions, including binary CE and categorical CE. We employ categorical CE in our implementation. This is because categorical CE works with multi-class as well as binary class and therefore is more flexible. Apart from the activation function and loss, another critical parameter is the learning rate (LR), which determines the number of steps the neural network takes during the learning process. If the LR is very large, the chances of divergence are high; if the LR is very small, we may become trapped in the local minima. Both of these situations are undesirable because they impair learning and, ultimately, model performance. Considering the importance of the LR in our implementation, we used an adaptive learning rate Adam (https://www.tensorflow.org/api_docs/python/tf/keras/optimizers/Adam (accessed on 28 September 2021)) that takes into account changing loss with an initial value of $1\times 10^{-3}$.

Overfitting is one of the major problems in deep learning. A similar instance was observed in our experiment, and therefore, to alleviate the overfitting, we used dropout in our implementation. Dropout is a well-established technique for restraining overfitting and functions similarly to a switch, turning off the neurons [67]. In addition to dropout, we also used the early stopping technique to monitor the validation accuracy. Early stopping is a very effective and simple form of regularisation [68]. Early stopping is a technique that stops the training once the monitored metric has stopped improving (https://www.tensorflow.org/api_docs/python/tf/keras/callbacks/EarlyStopping (accessed on 1 October 2021)). Furthermore, due to the sparsity and rapidly growing number of parameters that may be required, learning on a graph can be a difficult task. Therefore to reduce complexity, we enabled parameter sharing in our implementation by setting num_bases to a non-zero value. The training was then carried out for 500 epochs.

## 5. Performance Evaluation

This section contains details about the system and the software used in the experiment. Section 5.1 discusses the system setup, providing details about the system’s use, such as the number of Graphics Processing Units (GPUs). Section 5.2 provides information on training and testing. Section 5.3 provides information on the evaluation metrics and the reasons why we considered those evaluation metrics. Finally, in Section 5.4, we present our experimental results and their comparison to H2O, which we considered as a baseline. We chose H2O as a baseline because H2O is considered the industry-standard ML framework and is widely used in different industrial use cases (https://www.h2o.ai/solutions/#use-cases). In addition to comparison with the H2O, we also compare results with state of the art studies. The selection of the studies to compare were made based on the used similar evaluation metrics and the recency (2018 and later) of the article.

### 5.1. System Setup

We utilised Lambdalabs (https://lambdalabs.com/service/gpu-cloud (accessed on 27 September 2021)) GPU cloud. Our system consists of two NVIDIA RTX A6000 (https://www.nvidia.com/en-us/design-visualization/rtx-a6000/ (accessed on 27 September 2021)) GPUs, each GPU consisting of 48 GB GDDR6 memory, 200 GB Random Access Memory (RAM), and 1 TB secondary storage. Further, it consists of 28 Virtual Central Processing Units (VCPUS). The GPUs are linked together using NVIDIA NVLink (https://www.nvidia.com/en-us/data-center/nvlink/ (accessed on 2 October 2021)), a high-speed direct GPU-to-GPU interconnect, which improves the performance by allowing faster data exchange.

Most of the ML algorithms can be implemented using a variety of programming languages and libraries, such as Python (https://www.python.org (accessed on 2 September 2021)), R (https://www.r-project.org (accessed on 2 September 2021)), and Tensorflow ( https://www.tensorflow.org (accessed on 2 September 2021)). Our implementation uses Python version 3 and the StellarGraph [64] Python based ML library for graph-based ML, which is built on Tensorflow version 2. Further, we use the CUDA Compute Unified Device Architecture CUDA) (https://developer.nvidia.com/cuda-zone (accessed on 7 September 2021)) (version 11.2) parallel computing platform and programming model developed by NVIDIA for computation of the GPUs. During our initial experiment phase, we also used the LEO4 (https://www.uibk.ac.at/zid/systeme/hpc-systeme/leo4/ (accessed on 7 September 2021)). LEO4 is a high-performance compute cluster operated by the ZID (IT-Center) at the University of Innsbruck in close collaboration with the Research Area “Scientific Computing”.

### 5.2. Training and Testing

The training and testing of RGCN were performed using the Lambdalabs cloud. The details of the used Lambdalabs cloud system are provided in Section 5.1. The training of the RGCN model was performed for 500 epochs with 70% of the training data. Furthermore, during the training, different strategies such as dropout and early stopping were applied, which are discussed in detail in Section 4.4. The testing was performed using 30% of the data. Similar to RGCN, we used the Lambdalabs cloud for experiments using H2O. During the initial stage of the experiment, we also used the LEO4 cluster. The H2O training was conducted using 70% of the data similar to RGCN. Various techniques, such as k-fold cross validation, were employed to control overfitting during the training. The rest was used for testing (15% for validation and 15% for testing). Timing-wise, RGCN training took around 2 h, while H2O AutoML training took around 2.5 h. In terms of testing, the amount of time spent on prediction was negligible in comparison to the amount of time spent on training. The details on training, such as the used hyperparameters, their initial values, and the obtained tuned value after training, were presented in Section 4.3.

### 5.3. Evaluation Metrics

Evaluation metrics in ML help to understand how accurate a model prediction is and indicate how well the model is likely to perform in an unanticipated scenario. In ML, various evaluation metrics such as mean absolute error, mean squared error, accuracy, and recall exist, and thus selecting an appropriate metric is important. This is because other factors, such as data imbalance, must also be considered. Further, studies [69,70] have shown that relying on a single evaluation metric is not a good idea, especially in the case of highly skewed data. For example, one can achieve high accuracy by simply predicting the dominant-negative class. Despite the model’s high accuracy, we are more likely to predict negative for the positive class [70]. Such cases are extremely undesirable and necessitate the use of additional metrics. Therefore, in our study, we have considered four evaluation metrics: (i) accuracy, (ii) precision, (iii) recall, and (iv) Adjusted F-measure (AGF). Accuracy, the first evaluation metric, is the most common and is a widely used metric that measures the overall correctness of the model. Accuracy can be calculated by dividing the total correct predictions by the total predictions, both correct and incorrect using Equation (2). The other metric, precision measures the model’s exactness, while the recall measures evaluate the model effectiveness on the positive/minority class by measuring the accuracy of positive cases [69]. Equation (3) can be used to calculate the precision, and Equation (4) for the recall. The F1 score, which is the harmonic mean of precision and recall, explains how well precision and recall are balanced. However, studies have shown that the F1 score (or F1 measure) does not perform well in the case of highly unbalanced data [69]. Therefore, we considered the additional metric—AGF. The AGF metric is an improved version of the F1 score, which can perform well even in case of unbalanced data and thus tell us how well our model performs [69]. AGF can be calculated using Equations (5)–(7).

(2) $$Accuracy\;=\frac{TP\;+\;TN}{TP\;+\;TN\;+\;FP\;+\;FN}$$

(3) $$precision\;=\frac{TP}{TP\;+\;FP}$$

(4) $$recall\;=\frac{TP}{TP\;+\;FN}$$

(5) $$F_{2}=\;5\times \frac{precision\;\times \;recall}{(4\;\times \;recall)\;+\;precision}$$

(6) $$InvF_{0.5}=\frac{5}{4}\times \frac{precision\;\times \;recall}{(0.5^{2}\;\times \;recall)\;+\;precision}$$

(7) $$AGF\;=\sqrt{InvF_{0.5}\times \;F_{2}}$$

### 5.4. Results and Discussion

**Figure 10:**
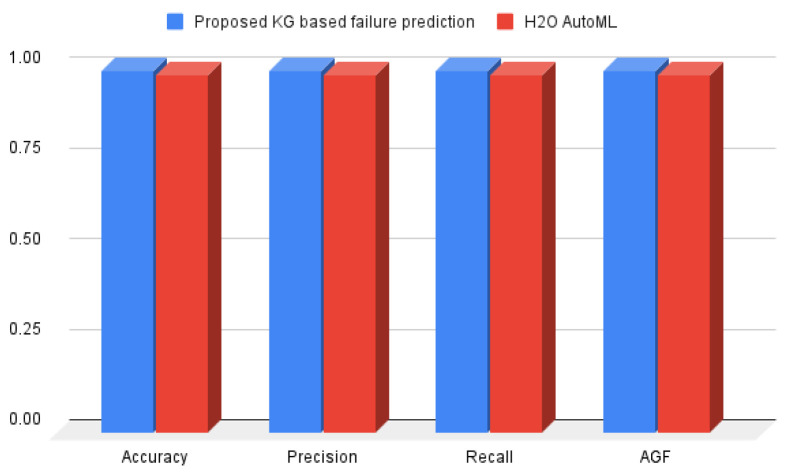
Proposed method versus the state of the art H2O result.

Figure 10 shows the experiment result. This includes our result from the cutting-edge H2O AutoML framework and the proposed method. Further, the evaluation metrics discussed in Section 5.3 are included in the experiment result depicted in Figure 10. The Y-axis in Figure 10 represents the percentage, and the X-axis includes the evaluation metrics recall, precision, and accuracy. Overall, we found that both experiments have high accuracy, precision, and recall. On closer inspection, however, we can see a difference in the result. The proposed method outperforms H2O AutoML in terms of performance by 1% improving result from 99% (baseline H2O AutoML) to 100%. In terms of accuracy, the proposed method, KG-based HD failure prediction, outperforms H2O by 1%. The same is true for precision and recall. We can observe from Figure 10 that the proposed KG-based HD failure prediction outperforms state of the art H2O by 1%. Further, in the case of the AGF, we observe a similar pattern to that of accuracy, precision, and recall. The high value of the AFG demonstrates that the result was not affected (or less affected) by the high-class imbalance.

In addition, we also compared our results with similar state of the art studies. Table 7 displays the findings from our studies as well as the compared relevant state of the art studies, which were chosen based on their recentness (2018 or later) and use of similar evaluation metrics. The best results were selected from the state of the art studies to compare. As can be seen from Table 7, the proposed KGs-based approach performs better despite our high data imbalance (with only 367 fail drives out of 13,553,809) compared to studies such as [49] (758 fail drives and 30,685 healthy drives for the first dataset and 47 fail drives and 7932 healthy drives for the second dataset).

In addition to comparing the accuracy, precision, and recall of our results, we also compared the training time of our solution to that of related work (see Table 7). As can be seen in Table 7, half of the studies we considered for comparison, with the exception of Züfle et al. [42], Han et al. [47], and Mamoutova et al. [24], did not report training time. Our experiment’s training time is significantly longer than Züfle et al. [42] and Han et al.’s [47] work. One of the reasons for the longer time, in our case, is the sample size. We used a sample size of 13,553,809, while Züfle et al. [42] used 68,411. The other reason is that the H2O AutoML approach performs extensive hyperparameter optimisation of multiple algorithms, whereas the KG-based approach requires learning relationships. Similarly, in the case of Mamoutova et al. [24], we observe a significant increase in our training time (compared to Mamoutova et al.’s [24] best of 183 s). However, when we compare our training time to the training time of algorithms such as LSTM, GRU, and LSTM with attention, which is approximately 4–7 h in Mamoutova et al.’s [24], our training time is shorter. Additionally, this demonstrates that training neural networks takes longer.

Finally, the results from our experiment and comparison to state of the art studies and H2O (baseline) substantiate our claim that using a KG can improve failure prediction. This improved failure prediction allows us to detect potential failures prior to their occurrence, allowing us to take proactive action and, as a result, improve the reliability.

## 6. Conclusions and Future Work

In this paper, we described a novel approach for predicting HD failures that combines ML and KGs. According to the evaluation results, combining these two technologies supports context-awareness and helps achieve higher accuracy of failure prediction in comparison to solutions that rely solely on ML. Moreover, the proposed approach can be applied to any other domain that requires prediction tasks, such as disease prediction (or classification). This generality of the presented approach is another benefit of our work. In addition, the use of KGs enables the incorporation of domain knowledge from domain experts and maintains human centricity, a step toward Industry 5.0 [71]. Since different domain experts may hold divergent views, incorporating domain knowledge may introduce bias, which can sometimes result in incorrect decision making. Solving such challenges would entail providing additional domain knowledge. The future work will focus on applying the proposed approach to other domains and industrial settings.

## Figures and Tables

**Table 1:** Summary of existing HD PdM solutions.

Study	Method		Performance	Training Time	Were SMART	Limitations
	ML	Semantics			Attributes Used?	
Mamoutova et al. [24]	✔	✔	Precision of 74%.	183 s to ~7 h, depending on the algorithm.	✖	The use of a semantic-only approach is restricted to predefined rules, such as the requirement that each fault be defined by a combination of values and monitoring parameters.
Su et al. [40]	✔	✖	Accuracy of 85.84%.	-	✔	
Shen et al. [41]	✔	✖	Failure detection rates of over 97.67%. False alarm rate of 0.017%.	-	✔	
Mashhadi et al. [45]	✔	✖	$R^{2}$ with less than 50%.	-	✔	
Han et al. [47]	✔	✖	Precision, recall, and F1-score are increased by 27.5–71.8%, 15.7–37.4%, and 26.8–53.2% respectively.	Training time is 10.6 s (0.6 s deviation).	✔	
Züfle et al. [42]	✔	✖	Accuracy, precision, and recall are 97.642%, 94.913%, and 96.97%, respectively.	The average training time of the multi-class classification approach is 174 s, while for the pre-filtering is 346 s.	✔	As previously stated, studies that rely solely on machine learning lack context awareness. Furthermore, the studies lack benefits such as the ability to incorporate expert knowledge and additional information, such as humidity, which is another reason for the failure associated with the use of KG, aside from the improved results.
Ganguly et al. [48]	✔	✖	Key performance indicators were established and used at different steps, such as design changes in production and pilot in test environment.	-	✔	
Liu et al. [6]	✔	✖	100.0% failure detection rate at a 0.02% false alarm rate.	-	✔	
Zang et al. [49]	✔	✖	Accuracy, precision, and recall are 92.6%, 89%, and 88.7%, respectively.	-	✔	
Santo et al. [50]	✔	✖	Accuracy of 98.45%, precision of 98.33% and recall of 98.34%.	-	✔	

**Table 2:** List of used SMART attributes and their definitions.

SN	SMART Attribute	Definition
1	Smart 5	Reallocated sector count.
2	Smart 10	Spin retry count.
3	Smart 187	Reported uncorrectable errors.
4	Smart 189	High fly writes.
5	Smart 190	Temperature difference or airflow difference.
6	Smart 198	Uncorrectable sector count.
7	Smart 197	Current pending sector count.
8	Smart 199	UltraDMA CRC error count.
9	Smart 188	Connection timeout.

**Table 3:** H2O stacked ensembles parameters.

Iterations	Regularisation	Predicators	Metalearner	Link	Lambda
99	Elastic Net (alpha = 0.5, lambda = $1.21\times 10^{-7}$)	3	GLM	logit	nlambda = 100, lambda.max = $1.298\times 10^{-4}$, lambda.min = $1.21\times 10^{-7}$, lambda.1se = $4.663\times 10^{-5}$

**Table 4:** GLM parameters.

Iterations	Regularisation	Predicators	Link	Lambda
45	Ridge (lambda = $8.302\times 10^{-8}$)	465	logit	nlambda = 30, lambda.max = 0.001141, lambda.min = $8.302\times 10^{-8}$, lambda.1se = $2.331\times 10^{-6}$

**Table 5:** DRF parameters.

Number of Trees	Min. Depth	Max. Depth	Min. Leaves	Max. Leaves
48	0	20	1	82

**Table 6:** XGBoost parameters.

Booster	Number of Trees	Learning Rate	Sample Rate	Max. Depth	Min. Rows	Tree Method
gbtree	99	0.3	0.6	10	5	hist

**Table 7:** Comparison with state of the art studies.

	Accuracy (%)	Precision (%)	Recall (%)	Training Time
Su et al.(2018) [40]	85.84	−	−	−
Zang et al. (2018) [49]	92.6	89	88.7	−
Santo et al. (2020) [50]	98.45	98.33	98.34	−
Han et al. (2020) [47]	−	71.8	37.4	10.6 s (streaming)
Züfle et al. (2020) [42]	97.642	94.913	96.97	174 s, on average
Mamoutova et al. (2021) [24]	−	74	−	183 s–7 h
Our baseline (H2O AutoML)	99	99	99	~2.5 h
Our proposed KG-based approach	100	100	100	~2 h

## Data Availability

Not Applicable.
